# Efficacy and safety of ozonated autohemotherapy in patients with hyperuricemia and gout: A phase I pilot study

**DOI:** 10.3892/etm.2014.1951

**Published:** 2014-09-09

**Authors:** LIAN-YUN LI, JIA-XIANG NI

**Affiliations:** Department of Pain Therapeutic Center, Xuanwu Hospital, Capital Medical University, Beijing 100053, P.R. China

**Keywords:** ozone, autohemotherapy, gout, hyperuricemia

## Abstract

Gout is a common form of arthritis; however, there are currently no effective therapies available. Ozonated autohemotherapy (O_3_-AHT) is a controversial, but successful method of treatment for a number of diseases. The present study is the first pilot study investigating the application of O_3_-AHT in patients with hyperuricemia and gout. In total, 10 patients diagnosed with gout were recruited and subjected to O_3_-AHT. Self-reported pain visual analog scale (VAS) scores and creatinine clearance values were evaluated prior to (T0), during (after the fifth session of O_3_-AHT treatment; 1–4 weeks; T1) and following the treatment course (5–28 weeks; T2). At T1, the creatinine clearance rate of the patients significantly increased from 105.14±35.33 (T0) to 121.45±44.52 ml/min (t=2.165, P=0.062), while the pain VAS score decreased from 5.35±2.78 (T0) to 3.30±2.21 (t=2.004, P=0.076). However, at T2, the creatinine clearance rate decreased slightly to 111.15±36.52 ml/min, and no statistically significant difference was observed from the value at T0 (t=1.723, P=0.123). The pain VAS score further decreased to 2.30±2.66 (t=2.628, P=0.027). In conclusion, O_3_-AHT decreased the creatinine clearance rate and the pain VAS scores of patients with hyperuricemia and gout; thus, may be a potential effective therapeutic approach.

## Introduction

Gout is a common form of arthritis. Elevated uric acid levels are observed in the blood of gout patients (hyperuricemia), resulting from the dysfunction of purine metabolism, increased uric acid formation and deposition. Other common gout characteristics are recurrent acute arthritis, tophus, joint deformity and gouty nephropathy, accompanied by hypertension, hyperlipemia, renal and cardiovascular diseases (1–3).

The worldwide prevalence of gout has doubled within the past three decades (4). It was reported that 6.1 million individuals were suffering from gout in America in 2010, constituting 1–2% of the adult population (5). In East China, the incidence rate was found to be 1.14% (6); thus, gout is becoming an increasingly important issue for public health.

Hyperuricemia is the most important risk factor in the development of gout, and epidemiological studies have demonstrated that the increasing incidence of gout is closely associated with diet, life style, medical care and lifetime extension (1,7–10). The Health Professionals Follow-up Study and National Health and Nutrition Examination Survey III revealed that dietary patterns had a significant influence on gout incidence (11,12). In particular, a high intake of meat, fish, beer and soft drinks was identified to be closely associated with increased rates of gout incidence, while a high intake of coffee, vitamin C and low-fat food was associated with reduced gout rates. Studies have suggested that excessive consumption of fructose also contributes towards an increased gout incidence. Although an unhealthy diet was the main cause of gout, genetic factors, renal function and medication, such as diuretics, also played a crucial role in disease development (3,13,14).

Almost all regular treatments administered to gout patients show side-effects and limitations. The use of non-steroidal anti-inflammatory drugs has been found to aggravate renal failure (15,16), hypertension and cardiovascular diseases (17) in gout patients, while glucocorticoid drugs have been shown to aggravate diabetes and hyperlipemia (18). In spite of the adverse effects of these drugs, long-term use remains very common for the treatment of gout. An effective gout therapy to replace the traditional treatment methods has yet to be developed, and efficient clinical recommendations for gout treatment are also unavailable (19).

Ozonated autohemotherapy (O_3_-AHT) is a controversial, but successful method of treatment for a number of diseases. To date, various studies have demonstrated that O_3_-AHT exhibits beneficial effects as an adjuvant therapy in patients with hepatitis B (20), diabetes, degenerative eye disease, complex regional pain syndrome, ischemic peripheral vascular disease (21) and osteonecrosis of the jaw (22,23). However, studies reporting the role of ozone in the therapy of gout are rare. In a preliminary study, O_3_-AHT was demonstrated to alleviate the pain of cancer patients, and during the treatment, a decline in the blood uric acid levels was detected. Based on these observations, a pilot study was designed to investigate the application of O_3_-AHT in gout patients.

## Materials and methods

### Patient characteristics

In total, 10 patients that had been diagnosed with gout were recruited for the study, including six males and four females, with ages ranging between 24 and 59 years. Two of the patients suffered from tophus, and three cases were diagnosed with acute gout attack. The clinical data were obtained from patient medical records. The study was approved by the Ethics and Academic Committees of the Capital Medical University (Beijing, China), and informed consent was obtained from all participants. The patients received standard O_3_-AHT at the Department of Pain Therapeutic Center of Xuanwu Hospital (Capital Medical University).

### Procedure

Screening criteria were based on the European League Against Rheumatism diagnosis standard for gout (24). The exclusion criteria comprised the main conditions known to change the concentration of uric acid in the blood: i) Deficiency of glucose-6-phosphate dehydrogenase; ii) severe allergic diseases; iii) use of angiotensin-converting enzyme inhibitors; iv) hyperthyroidism; v) thrombopenia; vi) severe cardiovascular diseases; vii) severe renal impairment (creatinine clearance rate of <30 ml/min); and viii) mental diseases. Patients who did not provide consent for participation in this trial were also excluded.

After screening, the 10 patients were treated with O_3_-AHT (1–4 weeks) and followed-up for 5–28 weeks. A commercially available ozone generator (Hyper Medozon Comfort; Herrmann Apparatebau GmbH, Kleinwallstadt, Germany) and Solar 8000M patient monitor (GE Healthcare, Pittsburgh, PA, USA) were used. During O_3_-AHT, 200-ml samples of the patient’s blood was mixed with 20 ml sodium citrate (3.8%), and exposed to an oxygen-ozone mixture with an ozone concentration of 50 μg/ml, for 5 min. Next, the blood was transfused back to the same patient. O_3_-AHT was performed three times a week, for a total of ten times for each patient.

### Data collection

Pain visual analog scale (VAS) scores (range, 0–10; a score of 0 indicated ‘no pain’ and higher scores indicate higher pain intensity) were reported for all the patients. Routine blood examinations (analyzing the count of leukocytes, erythrocytes, hemoglobin, platelets, neutrophils, neutrophil ratio, lymphocytes, lymphocyte ratio, mononuclear cells, eosinocytes and basophils) were performed for all the patients, as well as a comprehensive metabolic panel (analyzing the concentrations of serum creatinine, uric acid, high-density lipoprotein, low-density lipoprotein, total cholesterol, albumin, glucose and alanine aminotransferase). Immunological parameters (IgA, IgG, IgM, complement component 3 and complement component 4) were also examined. Data were collected prior to (T0), during (after the fifth session of treatment; 1–4 weeks; T1) and following the full course of treatment (5–28 weeks; T2).

### Statistical analysis

Statistical analyses were performed using SPSS software, version 17.0 (SPSS, Inc., Chicago, IL, USA). Experimental data obtained for each patient are presented as the mean ± standard deviation. The Student’s t-test (for paired samples) was used when the variances of two normal distributions were assumed to be equal. Under non-normal distribution or unequal variance conditions, the Wilcoxon signed-rank test was performed. Due to the small sample size of this pilot study, P<0.1 was considered to indicate a statistically significant difference, in order to reduce the probability of type II error.

## Results

### General patient characteristics

All 10 patients completed the treatment protocol. Nine patients underwent 10 courses of O_3_-AHT, while one patient received seven courses of O_3_-AHT.

Four cases were diagnosed with obesity (body mass index, >28 kg/m^2^), and two cases were diagnosed with type II diabetes mellitus and renal dysfunction (creatinine clearance rate, <80 ml/min). Six patients had food or medicine allergies, and one case was diagnosed with hyperlipemia. Patients had not been routinely administered drugs that may have affected the level of uric acid for at least six weeks prior to and during the study. The average time from the first diagnosis of gout was 4.5 years (range, 1–7 years; Table I).

### Efficacy on the creatinine clearance rate and pain VAS scores

**Figure 1:**
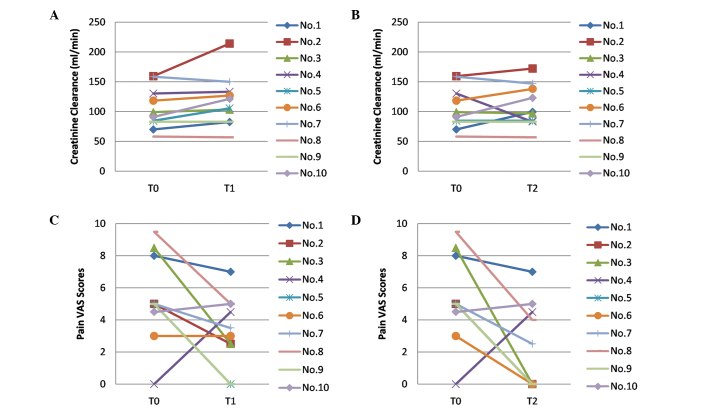
Changes in the creatinine clearance rate and pain VAS scores in patients with hyperuricemia and gout receiving ozonated autohemotherapy (O_3_-AHT). Comparison of the creatine clearance rates between (A) T1 vs. T0 and (B) T2 vs. T0. Comparison of the pain VAS scores between (C) T1 vs. T0 and (D) T2 vs. T0. VAS, visual analog scale; T0, prior to treatment; T1, following the fifth session of treatment; T2, following the course of O_3_-AHT treatment.

In general, a complete clinical response with an increased creatinine clearance rate and decreased pain VAS scores was achieved for all the patients (Table II; Fig. 1).

The creatinine clearance rate, which normally ranges between 80 and 120 ml/min, was determined to be 105.14±35.33 ml/min prior to O_3_-AHT (T0). After the fifth session of O_3_-AHT (T1), the creatinine clearance rate increased to 121.45±44.52 ml/min, which was significantly higher compared with the value at T0 (t=2.165, P=0.062). However, after the course of O_3_-AHT (T2), the creatinine clearance rate decreased slightly to 111.15±36.52 ml/min, and exhibited no statistically significant difference with the creatinine clearance rate at T0 (t=1.723, P=0.123).

Pain VAS scores are one of the most commonly used measures of pain intensity. At T0, the patients showed a mean pain VAS score of 5.35±2.78. The mean pain VAS score decreased to 3.30±2.21 at T1, which was significantly lower than the score at T0 (t=2.004, P=0.076). The mean VAS score further decreased to 2.30±2.66 at T2, and a statistically significant difference was observed when compared with the pain VAS score at T0 (t=2.628, P=0.027).

### Safety

Patients with hyperuricemia and gout showed good tolerance to the O_3_-AHT. No serious adverse reactions, including a rash, low blood pressure, abnormal liver function and abdominal pain, or acute gout attacks were observed during the treatment course. One patient developed mild dizziness and nausea during the seventh treatment session; however, normality was regained after 2 h.

## Discussion

Ozone is a gas found naturally in the Earth’s atmosphere, but can be produced from oxygen for medical use. Ozone exhibits wound-healing and antimicrobial properties, which may promote tissue repair and regeneration (25). Reactive oxygen species (ROS) and lipid oxidation products (LOPs) generated from the acute oxidative stress reaction of water, ozone and serum antioxidants have been reported to promote red blood cells to provide oxygen to ischemic tissues, resist viral infections by activating the immune system and release protective factors, including nitric oxide, carbon monoxide and platelet-derived growth factor (22,25,26).

O_3_-AHT is a new complementary therapeutic technology, during which physicians inject medical grade ozone gas into blood collected from a patient, and subsequently intravenously transfuse the blood back to the same patient. O_3_-AHT has recently developed into a modern medical approach that is widely used in the treatment of hepatitis B, diabetes and numerous other diseases (22). However, the efficacy and safety of O_3_-AHT in hyperuricemia and gout treatment remains unknown.

Low-dose ozone treatment has been universally accepted as a treatment for lumbar disc herniation, arterial atherosclerosis, ischemic cerebrovascular disease and a number of other diseases. Accurate measurements of ozone using a spectrophotometer have revealed that an ozone concentration of 20–80 μg/ml is not toxic. A preliminary clinical study identified that O_3_-AHT using this dose appeared to have an effect on pain alleviation in cancer patients. In addition, during this treatment, a decrease in the level of uric acid in the blood was observed, indicating a therapeutic potential for the application of O_3_-AHT in cases of hyperuricemia and gout. To explore the safety and efficacy of O_3_-AHT, a phase I clinical trial was performed in patients with hyperuricemia and gout.

In the present study, the creatinine clearance rate of patients receiving O_3_-AHT increased significantly, reaching values even beyond the reference range. Self-reported pain VAS scores were also significantly decreased after five sessions of O_3_-AHT, and were further decreased by 50% following completion of the treatment course. These results indicated that O_3_-AHT may be a potential complementary medical approach for patients with hyperuricemia and gout.

Neutrophils and macrophages are the main inflammatory cells in the pathological process of gout. It has been reported that monosodium urate causes the death of neutrophils and the release of lysozyme, and its metabolism may be influenced by the ROS and LOPs generated from the acute oxidative stress reaction resulting from O_3_-AHT (27–29). However, the exact mechanisms require further research.

However, limitations of the present study also require addressing. Firstly, the study involved a small group of patients; therefore, further studies involving more patients are required in order to confirm the observations. Secondly, a double-blind randomized controlled trial, which may provide further evidence, was unable to be performed due to the small study size population. Finally, in the current study, only the potential therapeutic role of O_3_-AHT was addressed; thus, the exact mechanisms and pathways require further elucidation. However, as a pilot clinical study, the present study has provided the basics for further research into the therapeutic potential and molecular mechanisms of O_3_-AHT in patients with hyperuricemia and gout.

In conclusion, 20 μg/ml ozone was identified to be an effective biological dose for O_3_-AHT, achieving a good curative effect and safety in patients with hyperuricemia and gout. Thus, low-dose O_3_-AHT may be a potential effective approach for hyperuricemia and gout patients. Further studies with a larger sample size, as well as investigations into the underlying mechanisms of O_3_-AHT, are required in the future.

## Figures and Tables

**Table I:** Patient demographics and baseline disease characteristics.

No.	Age (years)	BMI (kg/m^2^)	Creatinine clearance (ml/min)	Onset and region of gout	Tophus	Pain VAS score	Combined diseases
1	43	26.57	69.91	2005, right meta- tarsophalangeal joint	+	8	Noninfectious periodic fever syndrome
2	24	34.29	159.38	2010, right ankle joint	−	5	Allergic to shrimp; family history of gout
3	39	24.62	98.88	2006, right ankle joint	−	8–9	Allergic to seafood and erythromycin; history of hepatitis A
4	43	24.21	130.28	2007, general joint pain	−	0	Isn-Ab positive
5	43	31.74	84.42	2008, general joint pain	−	5	History of left knee surgery; sulfanilamide allergy
6	35	29.92	118.21	2009, left ankle joint	−	3	Rheumatoid factor-negative
7	33	32.72	158.23	2009, general joint pain	−	5	HBsAb-, HBeAb- and HBcAb-positive
8	54	26.12	57.82	2006, right toe joint	+	9–10	Type II diabetes; allergic to cold air
9	59	23.03	82.96	2005, left toe joint	−	5	NK cell count of 28%
10	40	27.64	91.02	2006, right toe joint	−	4–5	Type II diabetes

BMI, body mass index; VAS, visual analog scale; HBsAb, hepatitis B surface antibody; HBeAb, hepatitis B e antibody; HBcAb, hepatitis B core antibody; NK cells, natural killer cells; Isn-Ab, anti-insulin antibody.

**Table II:** Changes in the creatinine clearance rate and pain VAS scores in patients with hyperuricemia and gout treated with O_3_-AHT.

Time point	Creatinine clearance (ml/min)^a^	Paired t-test (vs. T0)	Pain VAS scores^b^	Paired t-test (vs. T0)
T0	105.14±35.33	-	5.35±2.78	-
T1	121.45±44.52	t=2.165	3.30±2.21	t=2.004
		P=0.062		P=0.076
T2	111.15±36.52	t=1.723	2.30±2.66	t=2.628
		P=0.123		P=0.027

a Creatinine clearance reference range, 80–120 ml/min;

b pain VAS score reference range, 0–10.

O_3_-AHT, ozonated autohemotherapy; VAS, visual analog scale; T0, prior to treatment; T1, following the fifth session of treatment; T2, following the course of O_3_-AHT treatment.
